# ceRNA Network Analysis Shows That lncRNA CRNDE Promotes Progression of Glioblastoma Through Sponge mir-9-5p

**DOI:** 10.3389/fgene.2021.617350

**Published:** 2021-03-09

**Authors:** Xiaobin Luo, Tianqi Tu, Yali Zhong, Shangyi Xu, Xiangzhou Chen, Ligang Chen, Fubing Yang

**Affiliations:** ^1^Department of Neurosurgery, The Affiliated Hospital of Southwest Medical University, Luzhou, China; ^2^Graduate School of Guizhou University of Traditional Chinese Medicine, Guiyang, China; ^3^Sichuan Clinical Research Center for Neurosurgery, The Affiliated Hospital of Southwest Medical University, Luzhou, China; ^4^Academician (Expert) Workstation of Sichuan Province, The Affiliated Hospital of Southwest Medical University, Luzhou, China; ^5^Laboratory of Neurological Diseases and Brain Function, The Affiliated Hospital of Southwest Medical University, Luzhou, China

**Keywords:** bioinformatics analysis, therapeutic targets, glioblastoma, competing endogenous RNA, mRNA-miRNA-lncRNA, CRNDE

## Abstract

Glioblastoma accounts for 45.2% of central nervous system tumors. Despite the availability of multiple treatments (e.g., surgery, radiotherapy, chemotherapy, biological therapy, immunotherapy, and electric field therapy), glioblastoma has a poor prognosis, with a 5-year survival rate of approximately 5%. The pathogenesis and prognostic markers of this cancer are currently unclear. To this end, this study aimed to explore the pathogenesis of glioblastoma and identify potential prognostic markers. We used data from the GEO and TCGA databases and identified five genes (*ITGA5*, *MMP9*, *PTPRN*, *PTX3*, and *STX1A*) that could affect the survival rate of glioblastoma patients and that were differentially expressed between glioblastoma patients and non-tumors groups. Based on a variety of bioinformatics tools for reverse prediction of target genes associated with the prognosis of GBM, a ceRNA network of messenger RNA (STX1A, PTX3, MMP9)-microRNA (miR-9-5p)-long non-coding RNA (CRNDE) was constructed. Finally, we identified five potential therapeutic drugs (bacitracin, hecogenin, clemizole, chrysin, and gibberellic acid) that may be effective treatments for glioblastoma.

## Introduction

Glioblastoma (GBM) is a primary tumor that occurs in the brain, and accounts for 45.2% of central nervous system tumors (Kanderi and Gupta, 2020). Ostrom et al. (2015) (Ostrom et al., 2015), 2008–2012) found that the incidence of GBM in Americans under the age of 54 was approximately 3.2/100,000, with the rate peaking at 75–84, representing a rate of approximately 15.24/100,000 people. Patients with GBM have poor prognosis, with a median survival time of approximately 15 months (Kanderi and Gupta, 2020). The 5-year survival rate after being diagnosed with GBM is approximately 5% (Alexander and Cloughesy, 2017). The main treatment methods for GBM include surgery, radiotherapy, chemotherapy, biological therapy, immunotherapy, and electric field therapy (Batash et al., 2017). Despite these multiple treatment methods, treatment outcomes are still unsatisfactory. The most fundamental reason for this is the unclear pathogenesis and prognostic markers of GBM. Therefore, this study aimed to explore the pathogenesis of GBM and identify potential prognostic markers.

Salmena et al. (2011) proposed that molecules can play the regulatory role of competing endogenous RNA (ceRNA) by competing with the same microRNA (miRNA) response elements (Salmena et al., 2011). The ceRNA hypothesis states that miRNAs can regulate multiple target genes, and the same target genes can be regulated by different miRNAs (Wang and Liu, 2017). One hypothesis of ceRNA suggests that lncRNAs can sponge and inactivate miRNAs, ultimately reducing mRNA degradation or silencing mRNA translation, thus affecting protein coding (Salmena et al., 2011). And the ceRNA hypothesis also suggests that the expression levels of lncRNA and miRNA are negatively correlated with each other and positively correlated with the expression of mRNA (Li et al., 2020). Based on the above hypothesis, we performed the construction of this network. The ceRNA network includes mRNAs, miRNAs, and long non-coding RNAs (lncRNAs). mRNA is a common protein-coding gene, with approximately 20,916 genes in the Genecards database^1^. Both miRNAs and lncRNAs belong to non-coding RNAs (ncRNAs), and there are approximately 219,587 genes in the body. miRNAs are RNA molecules that do not encode proteins and are approximately 22 bases in length. They negatively regulate gene expression by targeting specific mRNAs and ultimately induce their degradation or inhibit their translation (Bartel, 2004; Ballantyne et al., 2016). LncRNAs are RNA molecules that cannot encode proteins and are over 200 bases in length. Their presence in cells is low, but they have high specificity. The transcript of lncRNA is incompatible with classical mRNA has significant similarity (Derrien et al., 2012; Nakagawa and Kageyama, 2014). LncRNAs can function as ceRNAs, and can bind to miRNAs or mRNAs to isolate miRNA from their target mRNAs, thereby inhibiting miRNA (Yoon et al., 2014). The role of ceRNA in GBM is still unclear. In this study, we analyzed the ceRNA network to strengthen our understanding of the pathogenesis of GBM.

**FIGURE 1:**
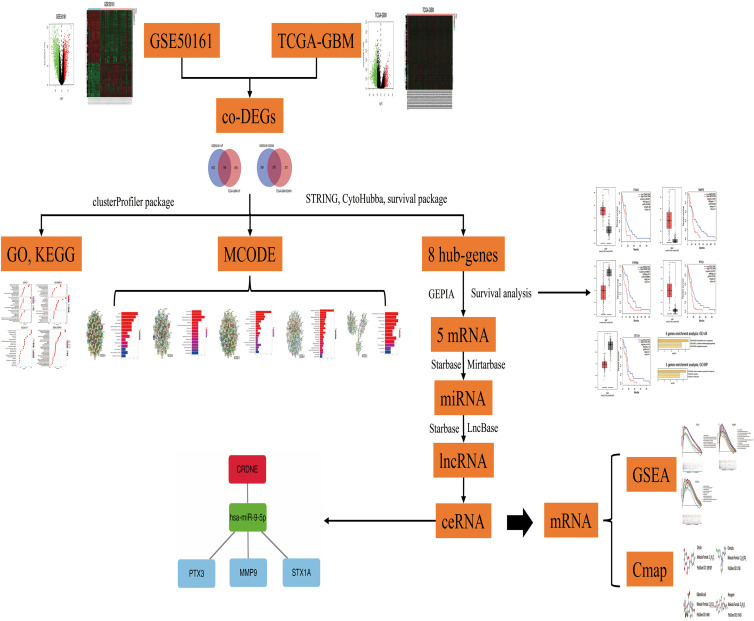
Research flow chart.

This research used bioinformatic tools to construct a ceRNA regulatory network and successfully predicted therapeutic drugs for GBM based on potential target genes. We believe that this study will improve our understanding of the pathogenesis of GBM and provide new insights into the treatment of this cancer. And the article research process is shown in Figure 1.

## Materials and Methods

### Microarray Data Analysis and Screening of Differentially Expressed Genes

To identify genome-wide gene expression datasets that have compared gene expression between GBM and normal tissues, we searched the widely used Gene Expression Omnibus (GEO^2^) database. The GSE50161 dataset (including 34 tumor samples and 13 non-tumorous samples) was selected for subsequent analyses. To improve the reliability of our differential mRNA screening results, we used GBM samples from the TCGA database^3^. Differentially expressed genes (DEGs) in the GSE50161 microarray were screened using the limma package^4^ in the R software^5^ (Ritchie et al., 2015). The cut-off conditions were set to an adjusted *P*-value <0.05, and the absolute value of log-fold change | log2FC| ≥ 2. Similarly, in the TCGA dataset, | log2FC| ≥ 2 and adjusted *P* < 0.05 were defined as statistically significant for the DEGs. A Venn diagram of the DEGs was constructed using the online tool ‘Calculate and draw custom Venn diagrams’^6^, and the common DEGs were identified.

### Functional Enrichment Analysis, Interaction Network Analysis and Hub Gene Identification

To further clarify the potential functional annotation and pathway enrichment associated with the DEGs, Gene Ontology (GO) analyses, including biological process (BP), cellular component (CC), molecular function (MF), and Kyoto Encyclopedia of Genes and Genomes (KEGG) pathway analyses were performed using the clusterProfiler package (version: 3.18.0)^7^ (Yu et al., 2012), with *p* < 0.05 representing statistically significant differences. The protein-protein interaction (PPI) network of DEGs was constructed using the online database Search Tool for the Retrieval of Interacting Genes (STRING, version: 11.0)^8^, and a confidence score of ≥0.4 was set as the threshold. Protein nodes that did not interact with other proteins were removed. Furthermore, the PPI network was analyzed to screen the significant modules and hub genes, using Cytoscape (version: 3.8.0)^9^ software (see text footnote 8) (version 3.7.2). The MCODE (version: 2.0.0)^10^ plug-in was used to select significant clustering modules based on the criteria: MCODE score >10 and number of nodes >20, and pathway enrichment analyses of the genes in these modules were performed using the clusterProfiler package (Bader and Hogue, 2003). Subsequently, the CytoHubba (version: 0.1)^11^ plug-in was used to screen the PPI network, and genes with degree >10 were identified as hub genes in the GBM (Chin et al., 2014).

### Survival Analysis and Verification

To further evaluate the prognostic value of hub genes in GBM, survival analyses were performed using the survival package (version: 3.2-7)^12^ (Therneau and Grambsch, 2000), using the default parameters, with the median set as the cut-off value. The GBM sample was selected as the dataset, and the hazard ratio (HR) was calculated based on both the Cox proportional hazards model and the Kaplan-Meier model. *P* < 0.05 was considered statistically significant. The GEPIA^13^ database includes RNA sequencing expression data of 9,736 tumors and 8,587 normal samples from TCGA and GTEx projects (Tang et al., 2017). Using this database to analyze the above gene survival, we set Group Cutoff to Quartile, Cutoff-High to 75%, Cutoff-Low to 25, and 95% Confidence Interval to NO. All parameters were set to default values to explore differential expression between tumor samples and normal samples, and perform differential expression analysis at the same time. *P* < 0.05 was considered statistically significant. To explore the role of these genes in the pathway, the metascape online analysis tool^14^ was used for pathway analysis (Zhou et al., 2019).

### ceRNA Network Construction

The ceRNA network not only affects signal transduction but also participates in the initiation and progression of a variety of diseases (Ala, 2020). Since the mechanism of ceRNA in the occurrence and development of many diseases has become clear, but its role in GBM is still unclear, we conducted this analysis based on a hypothesis of ceRNA. However, since all previous studies have sequentially studied lncRNA, miRNA and mRNA, we found through reading the literature that li et al. have successfully constructed ceRNA networks by backward prediction and this provides a new idea to reveal the development of diseases. Therefore, we also used the same method to construct the ceRNA network about GBM. First, to improve the accuracy of prediction, the miRNA-targeting mRNA was predicted using the Mirtarbase (version: 8.0)^15^ and Starbase^16^ databases. Cytoscape software was then used to construct the mRNA-miRNA network, and its plug-in Cytohubba was used to identify the top highly connected miRNAs. In addition, to verify whether miRNAs in the network meet the ceRNA hypothesis of low expression and poor prognosis. Finally, Oncolnc^17^ was used to perform survival analysis on miRNAs. The miRNA expression profile provided by the Firehose database^18^ was used to analyze the gene expression of the identified miRNAs. Based on the miRNAs identified above, the miRNA-lncRNA interaction was predicted in the Starbase and LncBase Predicted v.2 databases^19^ (Paraskevopoulou et al., 2016) and the intersection taken. The ceRNA hypothesis suggests that lncRNA expression and miRNA expression levels are negatively correlated and positively correlated with mRNA expression. So, the GEPIA database was used for survival analysis and expression analysis of the identified lncRNAs. All analyses were considered statistically significant at *P* < 0.05.

### Enrichment Analysis of DEGs and Screening of Small Molecule Therapeutic Drugs

To explore the influence of gene expression on the pathway, GSEA-enrichment analysis was performed. First, we extracted the protein-coding genes of TCGA-GBM. We took the first 50% of the expression of five DEGs as the high expression group, and the last 50% as the low expression group. All mRNAs in TCGA were selected and the GSEA desktop version (version: 4.0.3^20^, version 4.0.3) was used for GSEA analysis. The selected DEGs were used for drug prediction in Cmap (Connectivity Map)^21^, which is the most comprehensive transcriptome database for drug interference treatment, and is commonlys used for exploring potential drugs for treating diseases. A negative connectivity score is considered to represent a potential therapeutic drug. Therefore, enrichment <−0.7 and *P* < 0.01 were used for screening. The PubChem^22^ database was used to retrieve the molecular structure of identified drugs.

## Results

### Identification of Differentially Expressed Genes

**FIGURE 2:**
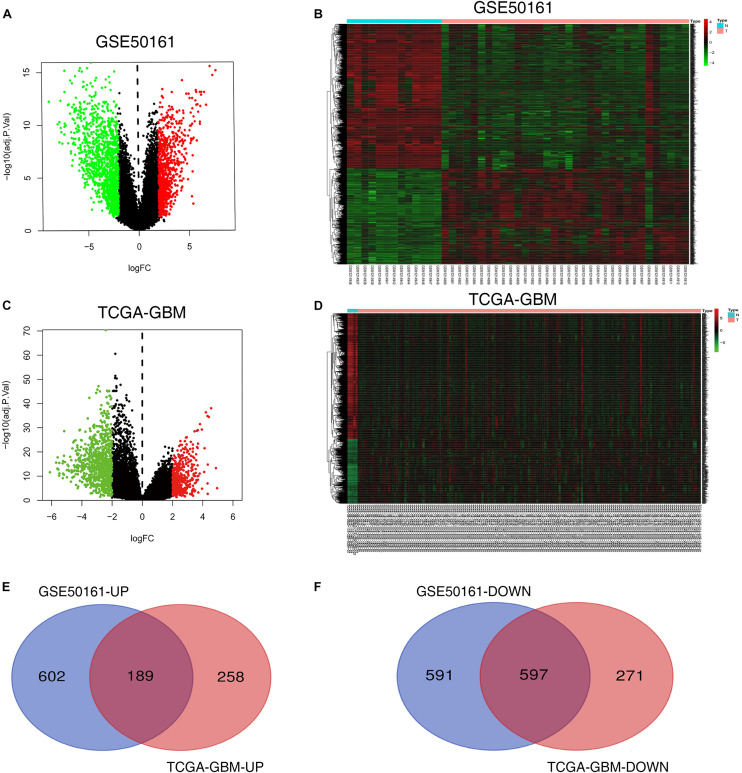
Screening of differentially expressed genes. **(A,B)** Volcano and heat maps of the GSE50161 dataset. **(C,D)** Volcano and heat maps of TCGA-GBM. **(E,F)** Common upregulated and downregulated DEGs in the GSE50161 and TCGA-GBM datasets. TCGA, The Cancer Genome Atlas Program; DEGs, differentially expressed genes; GBM, glioblastoma.

There were 1989 DEGs identified in the GSE50161 dataset, including 791 upregulated (UP) genes and 1198 downregulated (DOWN) genes. In the TCGA dataset, 1315 DEGs were identified, including 447 UP genes and 868 DOWN genes. The bioinformatics and evolutionary genomics webpage tool, Venn, was used to construct a Venn diagram of the two groups of DEGs. and 786 common DEGs were identified, including 189 common UP and 597 common DOWN genes (Figures 2A–F).

### Functional Enrichment Analysis of DEGs, Integration of Protein-Protein Interaction Network, and Module Analysis

**FIGURE 3:**
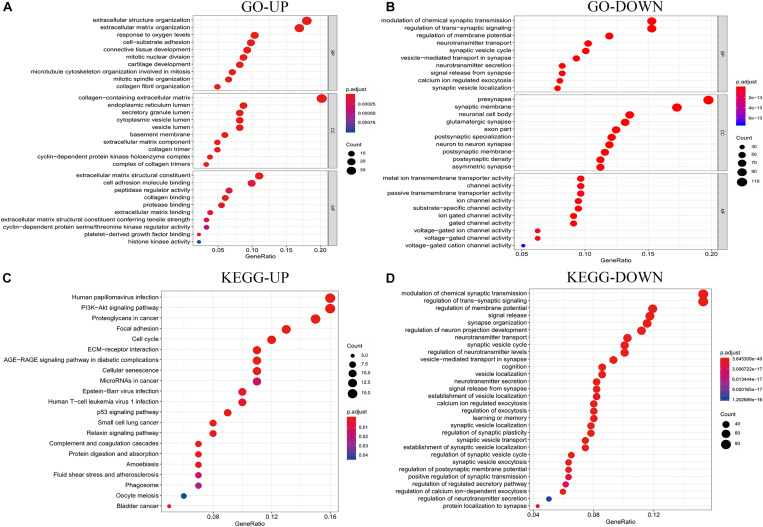
Analysis results of co-expressed genes in the GO and KEGG pathways. **(A,B)** Results of GO enrichment analysis of the DEGs that were common UP and DOWN genes. **(C,D)** Results of KEGG pathway analysis of the DEGs that are common UP and DOWN genes. DEGs, differentially expressed genes; GO, Gene Ontology; KEGG, Kyoto Encyclopedia of Genes and Genomes; UP, upregulated; DOWN, downregulated.

To further explore the biological function of the DEGs, the clusterProfiler package in R was used to perform GO and KEGG enrichment analyses. In the biological process (BP) group, upregulated DEGs were primarily enriched in extracellular structure organization, response to oxygen levels, and cell-substrate adhesion (Figure 3A). In contrast, downregulated DEGs were mostly enriched in the modulation of chemical synaptic transmission, regulation of trans-synaptic signaling, and regulation of membrane potential (Figure 3B). In the cellular component (CC) group, upregulated DEGs were primarily enriched in collagen-containing extracellular matrix, endoplasmic reticulum lumen, and cytoplasmic vesicle lumen (Figure 3A). In contrast, downregulated DEGs were mostly enriched in the presynapse, synaptic membrane, and neuronal cell body (Figure 3B). In the molecular function (MF) group, upregulated genes were primarily enriched in extracellular matrix structural constituent, cell adhesion molecule-binding, and peptidase regulator activity, while downregulated DEGs were mostly enriched in metal ion transmembrane transporter activity, channel activity, and passive transmembrane transporter activity (Figures 3A, 2B). Moreover, KEGG pathway analysis results showed that the upregulated DEGs were significantly enriched in the PI3K signaling pathway, cancer proteoglycans, focal adhesion, and cell cycle (Figure 3C). In contrast, downregulated DEGs were considerably enriched in the modulation of chemical synaptic transmission, regulation of trans-synaptic signaling, and regulation of membrane potential (Figure 3D).

**FIGURE 4:**
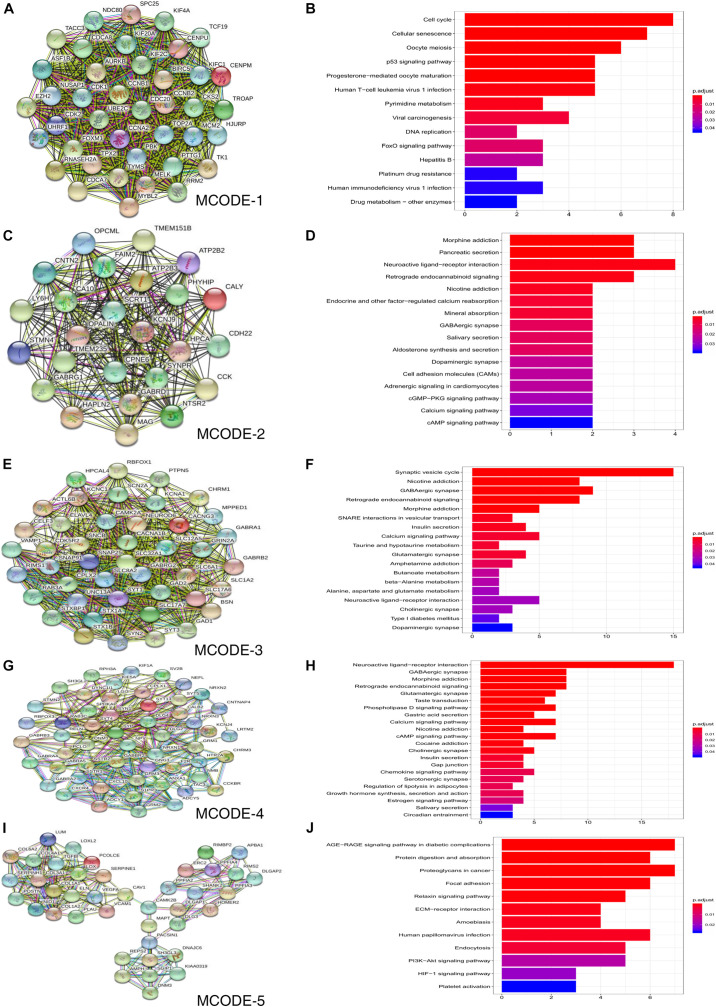
Results of MCODE analysis in common DEGs. **(A,C,E,G,I)** Use Cytoscape plugin MCODE to filter the important modules in DEGs, and then filter the final 5 important modules according to the filtering criteria. **(B,D,F,H,J)** Results of the 5 important modules pathway enrichment analyses. DEGs, differentially expressed genes.

The STRING online database was used to construct a PPI network consisting of 740 nodes and 7646 edges, and this network was then analyzed using Cytoscape software. Five significant clustering modules were selected using the MCODE plug-in, and the functional annotation of the DEGs involved in these modules was analyzed (Figure 4). Clustering module 1 consisted of 40 nodes and 713 edges, and the genes in module 1 were primarily associated with cell cycle, cellular senescence, oocyte meiosis, and the P53 signaling pathway (Figures 4A,B). Clustering module 2 consisted of 25 nodes and 274 edges. The genes in module 2 were primarily associated with morphine addiction, pancreatic secretion, and neuroactive ligand-receptor interaction (Figures 4C,D). Clustering module 3 consisted of 44 nodes and 489 edges, and the genes in this module were primarily associated with synaptic vesicle cycle, nicotine addiction, GABAergic synapse, and retrograde endocannabinoid signaling (Figures 4E,F). Clustering module 4, consisted of 54 nodes and 308 edges, with the genes in this module 4 beingprimarily associated with neuroactive ligand-receptor interaction, GABAergic synapse, and morphine addiction (Figures 4G,H). Clustering module 5, consisted of 41 nodes and 173 edges, had genes that were primarily associated with the AGE-RAGE signaling pathway in diabetic complications, proteoglycans in cancer, and focal adhesion (Figures 4I,J). A total of 408 DEGs were selected for further analysis using the degree algorithm in the CytoHubba plug-in.

### Survival Analysis and Verification

**FIGURE 5:**
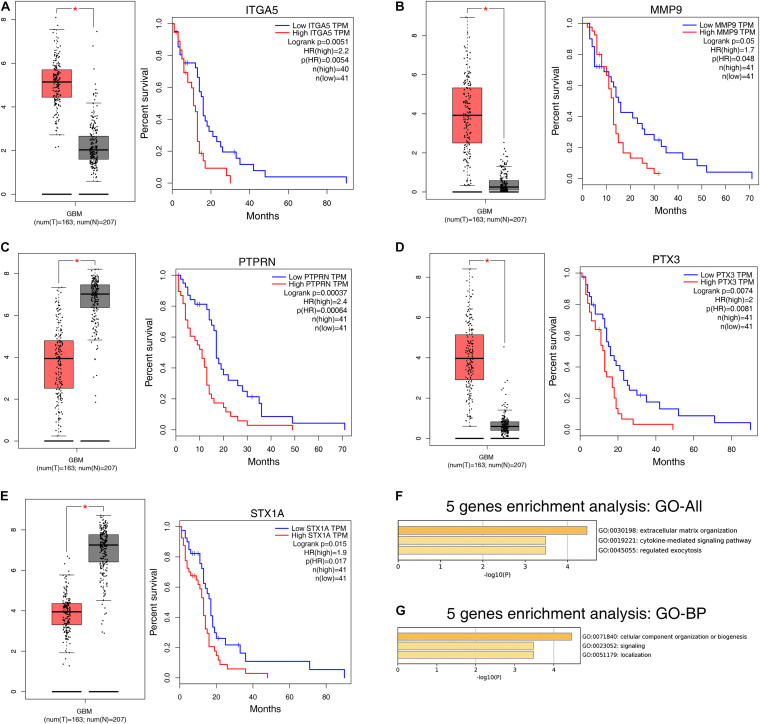
Survival analysis results of 5 DEGs. **(A–E)** Based on the GEPIA database for differential expression and survival analysis of the 5 selected target genes, it was found from the above images that these 5 genes were differentially expressed in normal and tumor samples, and significantly reduced the overall survival (OS) rate of patients. **(F)** Bar graph of enriched terms across input gene lists, colored by *P*-values. **(G)** The top-level biological processes can be viewed here. OS, overall survival; DEGs, differentially expressed genes.

The prognostic value of the 408 hub genes was evaluated using the survival package in R. Survival analysis revealed that most of them were not associated with the overall survival (OS) rate of patients with GBM. However, STX1A, MMP9, ITGA5, SYT5, PTPRN, IBSP, PTX3, and TIMP4 were significantly associated with the OS rate of patients with GBM based on both the Cox proportional hazards model and Kaplan-Meier model (Table 1). We identified five genes (*ITGA5, MMP9, PTPRN, PTX3*, and *STX1A*) that could significantly affect the OS rate of patients, and the difference between the tumor group and the normal group was statistically significant (Figures 5A–E). Using the Metascape database, we found that these five genes were concentrated in the extracellular matrix organization pathway, cytokine-mediated signaling pathway, regulated exocytosis in the GO pathway, and were mostly concentrated in ‘cellular component organization or biogenesis,’ ‘localization,’ and ‘signaling’ in the biological process group (Figures 5F,G).

**TABLE 1:** Survival analysis of all hub genes showed 8 genes with a prognostic value.

Gene	KM	HR	HR.95L	HR.95H	CoxP-value
STX1A	0.020011	1.034186	1.001931	1.06748	0.037591
MMP9	0.015394	1.005912	1.000337	1.011518	0.037637
ITGA5	0.041694	1.024932	1.007057	1.043124	0.006082
SYT5	0.045524	1.077953	1.008107	1.152638	0.028078
PTPRN	0.000968	1.055406	1.027832	1.083721	6.55E-05
IBSP	0.012715	1.009347	1.000701	1.018068	0.034033
PTX3	0.031197	1.008474	1.00371	1.013261	0.000478
TIMP4	0.035142	0.995475	0.992269	0.998691	0.005855

*STX1A, syntaxin 1A; MMP9, matrix metalloproteinase 9; ITGA5, integrin subunit alpha 5; SYT5, synaptotagmin 5; PTPRN, protein tyrosine phosphatase receptor type N; IBSP, integrin binding sialoprotein; PTX3,pentraxin 3; TIMP4, TIMP metallopeptidase inhibitor 4.*

### Construction of a Network of mRNA-miRNA-lncRNA

**FIGURE 6:**
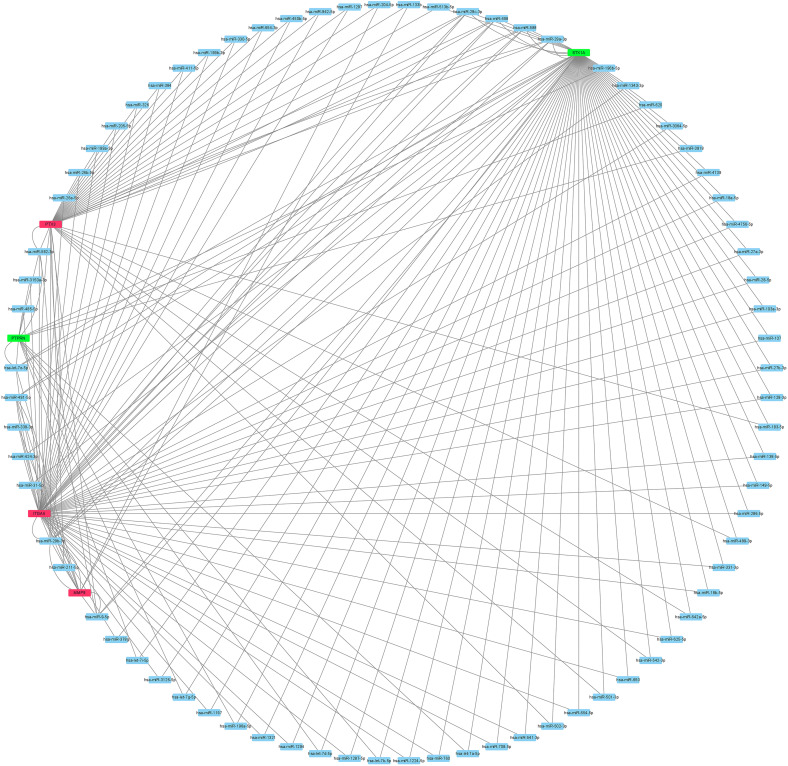
The network result of use mRNA to reverse-predict miRNA. Red represents highly expressed mRNA, green represents lowly expressed mRNA and blue represents miRNA. mRNA, messenger RNA; miRNA, microRNA.

**FIGURE 7:**
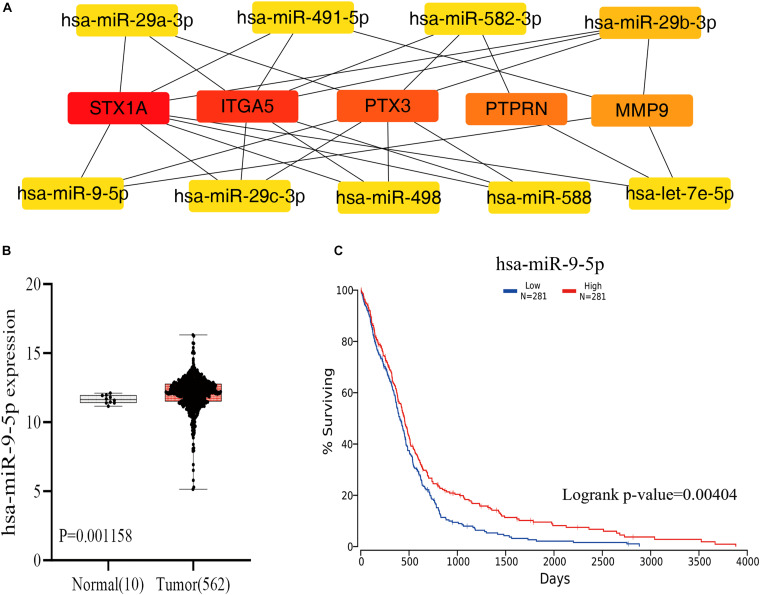
The plug-in Cytohubba of Cytoscape software was used to screen the miRNAs that were closely associated with the 5 mRNAs, through the visual analysis of the OncoLnc database, and one miRNAs were found to have a prognostic value. **(A)** Reverse prediction of miRNAs by 5 mRNAs and calculation of 9 miRNAs with the highest degree to 5 mRNAs using Cytoscape’s plugin Cytohubba. The yellow represents miRNA. **(B)** Results of differential analysis of miR-9-5p in normal tissue group and GBM group. **(C)** Results of miR-9-5p survival analysis in GBM. miRNA. mRNA, messenger RNA; miRNA, microRNA.

**FIGURE 8:**
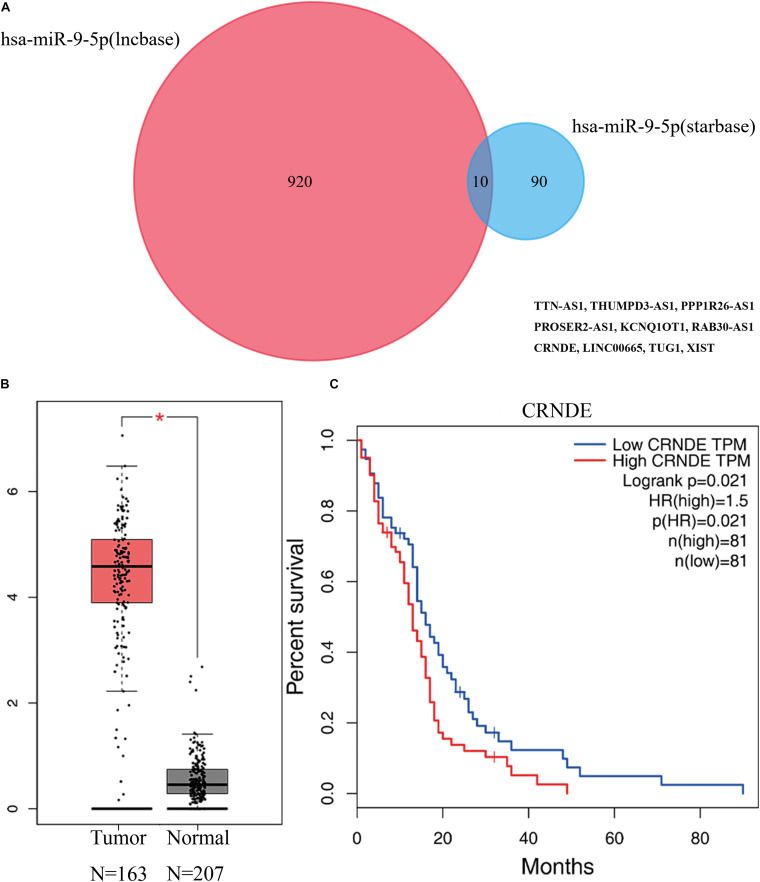
Prediction of lncRNA by miRNA, and differential expression and prognostic analysis using the GEPIA database showed that lncRNA CRNDE has prognostic value. **(A)** Results of miRNA reverse prediction lncRNA. **(B)** Results of differential analysis of lncRNA CRNDE in normal tissue group and GBM group. **(C)** Results of survival analysis of lncRNA CRNDE in GBM. miRNA. miRNA, microRNA; lncRNA, long non-coding RNA; GEPIA, Gene Expression Profiling Interactive Analysis.

Guided by the ceRNA hypothesis, we reverse predicted the miRNAs upstream of five mRNAs with prognostic value. The results found a total of 75 miRNAs targeting mRNA were found in the Mirtarbase and Starbase databases (Figure 6). Cytohubba identified the top nine highly connected miRNAs (Figure 7A; Table 2). The OncoLnc database identified one miRNA, hsa-miR-9-5p, which could significantly reduce the OS of GBM patients, and their differential expression in tumor and normal samples was apparent (Figures 7B,C). Nearly half of miR-9-5p in GBM group was lower than normal samples. Based on the above miRNAs, We used miRNAs to reverse predict their upstream lncRNAs that the miRNA-lncRNA network was constructed, and the intersection of the two databases was used to identifya total of 10 lncRNAs (TTN-AS1, THUMPD3-AS1, PPP1R26-AS1, PROSER2-AS1,KCNQ1OT1, RAB30-AS1,CRNDE, LINC00665, TUG1, and XIST) (Figure 8A). However, only CRNDE significantly reduced the OS of GBM patients (Figure 8B,C).

**TABLE 2:** The result of use mRNA to reverse-predict miRNA.

Name	Degree
hsa-miR-29b-3p	12
hsa-miR-582-3p	6
hsa-miR-491-5p	6
hsa-miR-29a-3p	6
hsa-miR-9-5p	6
hsa-miR-29c-3p	6
hsa-miR-498	6
hsa-miR-588	6
hsa-let-7e-5p	6

### Results of Enrichment Analysis and Small Molecule Therapeutic Drugs

**FIGURE 9:**
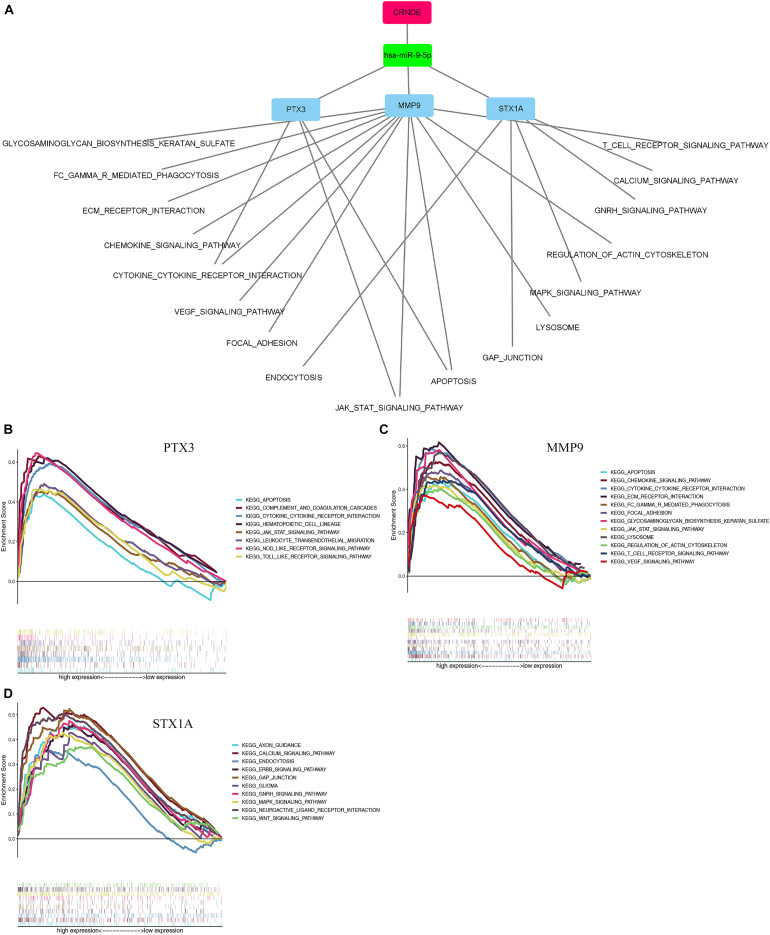
Single-gene GSEA enrichment results of 3 genes. **(A)** The results of using mRNA to stepwise reverse predict miRNA and LncRNA, and constructing a ceRNA network. The red rectangle represents key lncRNA. The green rectangle represents key miRNA. The blue rectangle represents key mRNA. **(B–D)** Single gene enrichment analysis of 3 mRNAs. GSEA, Gene Set Enrichment Analysis.

**FIGURE 10:**
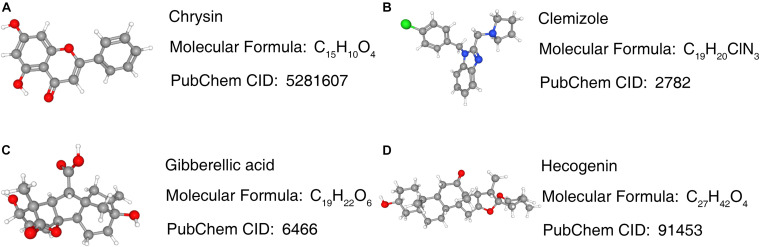
Prediction results of potential small molecule drugs for the treatment of GBM based on 3 target genes. **(A–D)** Prediction results of targeted drugs. GBM, glioblastoma.

The enrichment results of the three genes (STX1A, PTX3, MMP9) showed that they were significantly enriched in the JAK-STAT signaling pathway, apoptosis, and cytokine-cytokine receptor interaction pathways, which may be related to GBM invasion, metastasis, and adhesion (Figures 9A–D; Zhu and Li, 2011; Trejo-Solís et al., 2018; Henrik Heiland et al., 2019). Using the Cmap database, we used three mRNAs (STX1A, PTX3, MMP9) to predict potential therapeutic drugs for GBM. A total of five drugs, namely bacitracin (its structure has not been found yet), hecogenin, clemizole, chrysin and gibberellic acid were identified (Table 3; Figure 10).

**TABLE 3:** mRNA was used to predict potential drugs for the treatment of GBM.

Cmap Name	Mean	N	Enrichment	*P*	Specificity	Percent Non-Null
Bacitracin	−0.725	3	−0.866	0.00483	0.0182	100
Hecogenin	−0.752	4	−0.862	0.00066	0	100
Clemizole	−0.759	5	−0.848	0.00024	0	100
Chrysin	−0.754	3	−0.843	0.00773	0.0152	100
Gibberellic Acid	−0.652	4	−0.788	0.00408	0.0107	100

## Discussion

GBM is a malignant tumor that spreads rapidly in the central nervous system. Its prognosis is very poor and remains low even after treatment. Although the research on GBM has produced successful results, the pathogenesis of GBM is still unclear, and effective targeted drugs and prognostic markers are lacking. This study identified potential prognostic markers of GBM through integrated bioinformatics analysis, which are expected to provide new ideas and insights for the treatment of GBM.

Based on the ceRNA hypothesis, we gradually reverse predicted the upstream miRNA and lncRNA from mRNA, and finally successfully constructed the ceRNA network, in which all genes in the network are associated with poor prognosis of GBM. Importantly, our model fully conforms to the construction rules of the ceRNA network. The results of pathway enrichment analysis showed that PTX3 gene and MMP9 gene are jointly involved in JAK-STAT signaling, apoptosis and cytokine-cytokine receptor interaction pathways, and all the above pathways are related to the occurrence and development of GBM. The above pathways have been linked to the development of GBM.

The *ITGA5* gene belongs to the integrin alpha chain family. Integrin is a membrane protein composed of two subunits, which have cell adhesion and signal transduction functions (Desgrosellier and Cheresh, 2010; Xu et al., 2015). Feng et al. (2017) inhibited the expression of *ITGA5* gene to reduce the proliferation, invasion, and migration ability of GBM cells and found that it was potentially regulated by miR-330-5p, and found that the gene was highly expressed in GBM patients (*P* < 0.05), and was associated with poor prognosis in these patients (Figure 4A). MMP9 belongs to the matrix metalloproteinase (MMP) family. It is mainly involved in the degradation of the extracellular matrix, promotes remodeling of the extracellular matrix, and participates in angiogenesis (Long et al., 2017). Studies have found that the expression of MMP9 is an independent prognostic marker of primary GBM (Li Q. et al., 2016; Xue et al., 2017) and is related to the grading and aggressiveness of gliomas (Forsyth et al., 1999). They also found that this gene couldsignificantly reduce the survival rate of patients, which is consistent with the results of this study. Through single gene GSEA analysis, we found that MMP9 and PTX3 genes are involved in the JAK-STAT signaling, apoptosis, and cytokine-cytokine receptor interaction pathways. Overexpression of JAK-STAT signaling promoted the occurrence of immune escape in glioma (Chang and Lai, 2019). *PTPRN* is a member of the protein tyrosine phosphatase (PTP) family and an essential transmembrane protein of vesicles. It is related to the occurrence and progression of a variety of cancers (Frankson et al., 2017). Some studies have shown that it is negatively correlated with prognosis in GBM patients (Shergalis et al., 2018; Yin et al., 2019), which is consistent with our survival analysis (Figure 4C). *PTX3* is a component of humoral immunity, and it is a marker of inflammation (Locatelli et al., 2013). Studies have found that IL-1 induces the expression of PTX3, which is related to tumor invasion, migration, angiogenesis, and grade of GBM (Locatelli et al., 2013; Tarassishin et al., 2014). In addition, our study found that high PTX3 expression was associated with poor prognosis (Figure 4D). PTX3 and MMP9 are involved in a number of biological processes, which may be related to the occurrence and development of GBM. The alias of the *STX1A* gene, known as syntaxin 1A, is mainly involved in the contact of synaptic vesicles in the active area of neurons (Nakayama and Akagawa, 2017). Blocking the expression of *STX1A* using SNARE protein can reduce the invasiveness of GBM cells *in vitro* (Ulloa et al., 2015). We found that *STX1A* gene was downregulated in patients with GBM, with a hazard ratio of 1.9. Combined with patients’ survival curve, *STX1A* gene was found to be a poor prognostic marker in patients with GBM. In addition, enrichment analysis showed that these five genes were mainly enriched in the biogenesis, localization, and signaling pathways. We speculated that they might be related to the occurrence and development of GBM.

miRNAs are single-stranded non-coding RNAs, generally composed of 21–23 nucleotides (Tiwari et al., 2018). They are closely related to the growth and development of the body, and mainly regulate gene expression by inhibiting the translation of mRNA or promoting their degradation (Correia de Sousa et al., 2019). Under hypoxic conditions, miRNAs can promote cancer progression through editing (modification) (Eltzschig and Carmeliet, 2011). Several studies have found that miR-9 is mainly expressed in the nervous system and is associated with brain tumors. It can inhibit tumor development, and progress and differentiation (Coolen et al., 2013) (Cai et al., 2019). Zhang et al. (2019) found that miR-9-5p can downregulate the expression of FOXP2 and inhibit the proliferation of glioma cells. Our study found that the expression of mir-9-5p differed between the tumor and normal groups, and it can also be targeted and regulated by CRNDE genes. In addition, the expression pattern of mir-9-5p also conforms to the construction rules of the ceRNA network, which paves the way for further analysis.

lncRNA is a type of RNA molecule that is particularly associated with tumor growth, invasion, and metastasis (Chi et al., 2019). CRNDE is an lncRNA located on chromosome 16, which was first found to be upregulated in colorectal cancer (Graham et al., 2011). It is involved in a variety of biological processes in tumors, such as promoting proliferation, inhibiting cell apoptosis, inducing invasion and migration, and regulating inflammation, even with drug resistance and radiotherapy resistance (Lu et al., 2020). In recent years, it has been found to be a poor prognostic marker for liver cancer (Chen et al., 2016), lung cancer (Zhang et al., 2018), ovarian cancer (Szafron et al., 2015), and breast cancer (Fu et al., 2017). In addition, in glioma, the expression of the *CRNDE* gene is related to its growth and invasion (Wang et al., 2015). It is also an independent prognostic marker for gliomas (Jing et al., 2016). *CRNDE* was also found to regulate the occurrence and development of glioma through the TLR3-NF-κB-cytokine signaling pathway (Li et al., 2017). Zheng et al. found that CRNDE could inhibit miR-384 expression, leading to an increase in PIWIL4, causing an increase in the expression of its downstream product MMP9, with the end result of glioblastoma proliferation and migration (Zheng et al., 2016). The correlation between CRNDE gene and PTX3 and STX1A genes has not been correlated for the time being confirmation in the literature, but PTX3 has been shown to be associated with poor prognosis in gliomas (Locatelli et al., 2013), and in connection with our results, its upstream target marker may be the CRNDE gene. This study found that the *CRNDE* gene is highly expressed in GBM patients and is related to poor prognosis of patients. Based on the ceRNA hypothesis, the *CRNDE* gene can competitively bind to mir-9-5p, thus increasing the transcription levels of *PTX3, MMP9*, and *STX1A* regulated by mir-9-5p, thus affecting the JAK-STAT signaling pathway, apoptosis, and cytokine-cytokine receptor interaction pathways, and promoting the occurrence and development of GBM.

In this study, we identified the common DEGs through joint analysis of the GSE50161 dataset and GBM samples in the TCGA database, and then carried out a survival analysis. We identified five key mRNAs, which were significantly differentially expressed between GBM and normal samples, with HR values greater than 1. Together with the survival analysis curve, we confirmed that these were important factors affecting the prognosis of GBM patients. To explore the prognosis-related ceRNA network, we used five mRNAs to reverse predict miRNA, and then used miRNA to predict lncRNA. Finally, we successfully constructed the mRNA-miRNA-lncRNA ceRNA network. We also analyzed the single gene GSEA of the ceRNA network and found that PTX3 and MMP9 genes could target the JAK-STAT signaling, apoptosis, and cytokine-cytokine receptor interaction pathways, which were found to be associated with GBM. The ceRNA network suggested that the *CRNDE* gene may regulate the expression of critical genes through the sponge miR-9-5p. It also indicated that *CRNDE* may be a therapeutic target for GBM. By using key genes to predict potential drugs for its treatment, five drugs were identified, related studies confirmed that bacitracin, clemizole and chrysin can induce GBM cell apoptosis *in vitro* (Richter et al., 2014, 5; Li S. et al., 2016; Wang et al., 2018). Although there were no relevant studies on two of these (hecogenin and gibberellic acid).

## Conclusion

Through the integration of various bioinformatics analyses, we successfully constructed a reverse mRNA prediction model based on the hsa-mir-9-5p/STX1A, MMP9, and PTX3 ceRNA network, which can promote our understanding of the occurrence and development of GBM. It is important that all genes in the ceRNA network reduce the overall survival rate of GBM patients; HR values are greater than 1, suggesting that they are risk factors for GBM, so they can be used as therapeutic targets for GBM. GSEA analysis of a single gene showed that MMP9 and PTX3 genes may promote the occurrence and development of GBM through the JAK-STAT signaling, apoptosis, and cytokine-cytokine receptor interaction pathways. We also identified five potential drugs for the treatment of GBM, among which Bacillus, clemizole, and chrysin have been verified by relevant *in vitro* tests. However, there are currently no relevant tests for hecogenin and gibberellic acid, which indicates that our findings can provide ideas for future research.

Our study has some limitations. First, the sample size was small. To compensate for this, we used the GEO and TCGA databases to increase credibility. Second, all samples were from America. The samples from America may differ from GBM in the Chinese population. Third, there is no relevant basic experiment for detecting the expression levels of key genes in the ceRNA network in clinical samples. Finally, we are the first group to predict the ceRNA network through analysis of the reverse transcription of mRNA. However, only one link was predicted. ceRNA is very complex, and its complex regulatory network needs to be further clarified.

## Data Availability Statement

The original contributions presented in the study are included in the article/supplementary material, further inquiries can be directed to the corresponding author/s.

## Author Contributions

XL, TT, YZ, SX, XC, LC, and FY designed the study. XL, TT, YZ, SX, and XC performed the bioinformatics analysis and interpretation of the data. XL, TT, and YZ wrote the manuscript. LC and FY revised the manuscript and gave final approval of the version to be published. All authors read and approved the final manuscript.

## Conflict of Interest

The authors declare that the research was conducted in the absence of any commercial or financial relationships that could be construed as a potential conflict of interest.

## Footnotes

1 https://www.genecards.org/

2 www.ncbi.nlm.nih.gov/geo/

3 https://www.cancer.gov/about-nci/organization/ccg/research/structural-genomics/tcga

4 https://bioconductor.org/packages/release/bioc/html/limma.html

5 https://www.r-project.org/, version 4.0.0

6 http://bioinformatics.psb.ugent.be/webtools/Venn/

7 https://bioconductor.org/packages/release/bioc/html/clusterProfiler.html

8 http://string-db.org

9 https://cytoscape.org/

10 http://apps.cytoscape.org/apps/mcode

11 http://apps.cytoscape.org/apps/cytohubba

12 https://cran.r-project.org/web/packages/survival/index.html

13 http://gepia.cancer-pku.cn/index.html

14 http://metascape.org/gp/index.html#/main/step1

15 http://mirtarbase.cuhk.edu.cn/php/index.php

16 http://starbase.sysu.edu.cn/

17 http://www.oncolnc.org/

18 https://gdac.broadinstitute.org/

19 http://carolina.imis.athena-innovation.gr/diana_tools/web/index.php?r=lncbasev2%2Findex-predicted

20 https://www.gsea-msigdb.org/gsea/index.jsp

21 https://portals.broadinstitute.org/cmap/

22 https://pubchem.ncbi.nlm.nih.gov/
